# Coenzyme Q10 coadministration with diclofenac augmented impaired renal function in broiler chickens (*Gallus gallus domesticus)*

**DOI:** 10.14202/vetworld.2020.642-648

**Published:** 2020-04-10

**Authors:** Yasser Albadrany, Ahmed Naser

**Affiliations:** Department of Physiology, Biochemistry and Pharmacology, College of Veterinary Medicine, University of Mosul, Mosul, Iraq

**Keywords:** broiler, coadministration, COQ10, diclofenac, kidney liver

## Abstract

**Aim:**

This study aimed to investigate the effects of coenzyme Q10 (COQ10) and diclofenac coadministration on the hepatorenal function in broiler chickens (*Gallus gallus domesticus*).

**Materials and Methods:**

Birds (21 days old) were divided into six groups of eight birds each. The 1^st^ group was the control, the 2^nd^ group was treated orally with COQ10(30mg/kg b.wt), the 3^rd^and 4^th^groups were treated intraperitoneally with diclofenac sodium at doses 1 and 2mg/kg b.wt, respectively, and the 5^th^and 6^th^groups were treated with COQ10 (dose 30mg/kg b.wt, P.O.) and diclofenac sodium (dose 1mg/kg b.wt, I.P.) and COQ10 (dose 30mg/kg b.wt, P.O.) and diclofenac sodium (dose 2mg/kg b.wt, I.P.), respectively. The experiment lasted 5days. Twenty-four hours after the last administration, all the birds were sacrificed through cervical dislocation; blood samples were collected for serum biochemical analysis.

**Results:**

COQ10 induced a significant increase in aspartate aminotransferase (AST), urea, creatinine, sodium, potassium, and chloride, while diclofenac induced a significant increase in alanine aminotransferase (ALT), AST, total cholesterol, triglyceride, high-density lipoprotein, urea, creatinine, sodium, potassium, and chloride. However, when COQ10 and diclofenac were coadministered, we observed that COQ10 decreased the liver injury caused by diclofenac. However, COQ10 could not relieve the kidney injury caused by diclofenac, but worsened the impaired renal function.

**Conclusion:**

COQ10 protects the liver against diclofenac-induced liver injury while augmenting diclofenac-induced kidney injury.

## Introduction

Diclofenac is a nonsteroidal anti-inflammatory drug (NSAID) used clinically in the field of veterinary medicine as a painkiller, anti-inflammatory, and antipyretic [1]. NSAIDs inhibit the cyclooxygenase (COX) enzyme, which leads to reduction in prostaglandin synthesis [2]. The diminution of physiologically vital prostaglandins during chronic COX inhibition has momentous life-threatening side effects, including cardiovascular, gastrointestinal, and renal toxicity [3]. In addition, diclofenac has a toxic effect on the liver tissue (hepatotoxicity)[4]. Diclofenac is widely metabolized in the liver through two major pathways (hydroxylation and glucuronidation) in both humans and experimental animals[5]. Diclofenac has caused a large and rapid decline in the Indian subcontinent’s number of wild Gyps vultures [6], resulting from eating dead cattle treated with diclofenac. These wild birds are now at risk of extinction [7]. The scientists attributed the cause of high rates of losses among these birds to the high toxicity of diclofenac on the kidney tissue, represented by recent severe necrosis of cells lining renal tubules [8].

Coenzyme Q10 (COQ10) (2, 3-dimethoxy-5-methyl-6-decaprenyl benzoquinone) is a fat-soluble, vitamin-like quinone generally well known as CoQ, ubiquinone, and vitamin Q10 [9]. COQ10 is a coenzyme present in inner mitochondrial enzyme complexes and is associated with adenosine 5′-triphosphate (ATP) synthesis. It is endogenously synthesized in mammals. COQ10 has been reported to have strong antioxidant activity[10]. COQ10 is found not only to assist electron transport in ATP creation but also to donate to stabilization of mitochondrial permeability transition pore and guard against apoptosis and autophagy [11].

This study aimed to investigate the effects of COQ10 and diclofenac coadministration on the hepatorenal function in broiler chickens (*Gallus gallus domesticus*).

## Materials and Methods

### Ethical approval

The birds were handled according to the instructions by the animal ethics committee of the College of Veterinary Medicine of Mosul. The Scientific Committee of the Department of Physiology, Biochemistry, and Pharmacology of the Veterinary Medicine College, University of Mosul, reviewed and approved the protocol of this study.

### Experimental design

This study was conducted during the period of April 2018 to May 2018. Forty eight one-day-old broiler chicks were obtained from a local hatchery (Basheka company, Mosul, Iraq). These birds were reared in the experimental laboratory unit of the College of Veterinary Medicine of Mosul. All the birds were fed with commercial broiler feeds produced by the Alnebras Company (Mosul, Iraq). Feed and water were provided *ad libitum* during the experiment. After 21days, the birds were randomly divided into six groups as follows: Each group had eight broiler birds, and the treatment plan consisted of a single dose/day for 5 consecutive days.

Group1: Received distilled water (volume of injection 2mL/kg b.wt) through intraperitoneal injection (I.P.) and sunflower oil (volume of dose 5mL/kg b.wt) through oral route.

Group2: Received COQ10 (Premier Health Products Ltd., UK), does 30mg/kg b.wt, P.O.

Croup 3: Received diclofenac sodium (Gulf Pharmaceutical Industries, U.A.E.), dose 1mg/kg b.wt, I.P.

Group4: Received diclofenac sodium, dose 2mg/kg b.wt, I.P.

Group5: Received COQ10, dose 30mg/kg b.wt, P.O. and diclofenac sodium, dose 1mg/kg b.wt, I.P.

Group6: Received COQ10, dose 30mg/kg b.wt, P.O. and diclofenac sodium, dose 2mg/kg b.wt, I.P.

The experiment lasted 5days. Twenty-four hours after the last administration, all the birds were sacrificed by cervical dislocation procedure followed by blood collection for serum biochemical analysis (alanine aminotransferase [ALT], aspartate aminotransferase [AST], total cholesterol, triglyceride, high-density lipoprotein [HDL], urea, creatinine, sodium, potassium, and chloride) using Dri-Chem NX500 autoanalyzer (Fujifilm Corporation, Japan).

Very low-density lipoprotein (VLDL) cholesterol in serum and LDL cholesterol in serum were estimated by employing the Friedewald formula [12]. The outcome is expressed in mg/dL of serum.

VLDL = Triglycerides ÷ 5

LDL = Total cholesterol ‒ (VLDL + HDL).

### Statistical analysis

The data were statistically analyzed by one-way analysis of variance followed by the least significant difference test. The level of significance was p<0.05.

## Results

### Serum ALT

The results revealed a significant increase in serum ALT activity in samples from birds of the group treated with diclofenac (2mg/kg) as compared to the control group, while there was no significant difference in the enzyme activity in samples from birds of the groups treated with COQ10(30mg/kg), diclofenac (1mg/kg), diclofenac (1mg/kg) + COQ10(30mg/kg), and diclofenac (2mg/kg) + COQ10(30mg/kg) when compared with the control group. We observed a decrease in enzyme activity in the group treated with diclofenac (2mg/kg) + COQ10(30mg/kg), but the difference was not statistically significant as compared with the diclofenac (2mg/kg) group (Table-1).

**Table-1:** Effect of COQ10 and diclofenac on ALT and AST (n=8 birds).

Group	ALT (U/L)	AST (U/L)
Control	23.25±0.99^a^	100.87±2.97^a^
COQ10 (30 mg/kg)	23.37±0.67^a^	125.00±7.69^b^
Diclofenac (1 mg/kg)	23.75±0.59^a^	134.00±4.35^b,c^
Diclofenac (2 mg/kg)	26.12±0.71^b^	148.25±5.56^c,d^
Diclofenac (1 mg/kg) +COQ10 (30 mg/kg)	22.62±0.88^a^	147.25±5.57^c,d^
Diclofenac (2 mg/kg) +COQ10(30 mg/kg)	25.00±1.01^ab^	162.12±7.11^d^

Values in each column followed by different superscript letters are significantly different at 5% level of significance. ALT=Alanine aminotransferase, AST=Aspartate aminotransferase

### Serum AST

There was a significant increase in serum AST activity of the groups which treated with COQ10(30mg/kg), diclofenac (1mg/kg), diclofenac (2mg/kg), diclofenac (1mg/kg) + COQ10(30mg/kg), and diclofenac (2mg/kg) + COQ10(30mg/kg) when compared with the control group. The activity of the enzyme in the groups treated with diclofenac (2mg/kg), diclofenac (1mg/kg) + COQ10(30mg/kg), and diclofenac (2mg/kg) + COQ10(30mg/kg) was significantly increased when compared with the group treated with COQ10(30mg/kg). We also observed a significant increase in enzyme activity of the group treated with diclofenac (2mg/kg) + COQ10(30mg/kg) in comparison with the group treated with diclofenac (1mg/kg) (Table-1).

### Total cholesterol

We observed a decrease in total cholesterol concentration, but it was not significant in the group treated with COQ10(30mg/kg) as compared with the control group. There was a significant increase in total cholesterol concentration in groups treated with diclofenac (1mg/kg), diclofenac (2mg/kg), and diclofenac (2mg/kg) + COQ10(30mg/kg) as compared with the control group. The results revealed that there was no significant difference in total cholesterol concentration in the group treated with diclofenac (1mg/kg) + COQ10(30mg/kg) as compared with the control group and the group treated with COQ10(30mg/kg) (Table-2).

**Table-2:** Effect of COQ10 and diclofenac on lipid profile (n=8).

Group	Total cholesterol (mg/dl)	Triglyceride (mg/dl)	HDL (mg/dl)	LDL (mg/dl)	VLDL (mg/dl)
Control	138.72±4.19^a^	53.68±3.39^a^	27.16±1.28^a^	100.83±4.0^a^	10.73±0.67^a^
COQ10 (30 mg/kg)	127.50±4.54^a,b^	50.49±3.76^a^	26.29±0.81^a^	91.12±5.30^a,b^	10.09±0.65^a^
Diclofenac (1 mg/kg)	166.09±8.10^c^	121.16±4.9^b^	28.73±1.10^a^	113.12±9.5^a,c^	24.23±0.83^b^
Diclofenac(2 mg/kg)	178.84±6.29^c,d^	134.17±7.5^bc^	42.94±0.66^b^	109.07±7.2^a,c^	26.83±1.47^b,c^
Diclofenac (1 mg/kg)+COQ10 (30 mg/kg)	147.55±7.87^ac^	79.37±5.59^d^	39.36±1.62^c^	92.32±7.51^a,b^	15.87±1.11^d^
Diclofenac (2 mg/kg)+COQ10 (30 mg/kg)	156.76±9.02^c,d^	108.82±4.9^b^	39.61±1.06^c^	95.39±9.6^a,b^	21.76±0.97^b^

Values in each column followed by different superscript letters are significantly different at 5% level of significance. HDL=High-density lipoprotein, LDL=Low-density lipoprotein, VLDL=Very low-density lipoprotein

### Triglyceride

There was no significant difference in triglyceride concentration in the group treated with COQ10(30mg/kg) as compared with the control group. Diclofenac (1mg/kg) and diclofenac (2mg/kg) show increases in triglyceride concentration when compared with the control group and COQ10(30mg/kg) group. Furthermore, we observed a significant decrease in triglyceride concentration in the group treated with diclofenac (1mg/kg) + COQ10(30mg/kg) when compared with diclofenac (1mg/kg) and diclofenac (2mg/kg) groups. Furthermore, there was a decrease in the triglyceride concentration in the group treated with diclofenac (2mg/kg) + COQ10(30mg/kg), but there was no significant difference in comparison with diclofenac (1mg/kg) and diclofenac (2mg/kg) groups (Table-2).

### HDL

Serum HDL concentration in groups treated with COQ10(30mg/kg) and diclofenac (1mg/kg) showed no significant difference in comparison with the control group, but there was an elevation in serum HDL concentration in the group treated with diclofenac (2mg/kg) as compared with control, COQ10(30mg/kg), and diclofenac (1mg/kg) groups. Asignificant decrease in serum HDL concentration was observed in groups treated with diclofenac (1mg/kg) + COQ10(30mg/kg) and diclofenac (2mg/kg) + COQ10(30mg/kg) in comparison with the group treated with diclofenac (2mg/kg) (Table-2).

### LDL

Serum LDL concentration showed no significant difference in all treatment groups (COQ10(30mg/kg), diclofenac (1mg/kg), diclofenac (2mg/kg), diclofenac (1mg/kg) + COQ10(30mg/kg), and diclofenac (2mg/kg) + COQ10(30mg/kg) in comparison with the control group. There was also a significant decrease in serum LDL concentration of groups treated with COQ10(30mg/kg), diclofenac (1mg/kg) + COQ10(30mg/kg), and diclofenac (2mg/kg) + COQ10(30mg/kg) in comparison with diclofenac (1mg/kg) and diclofenac (2mg/kg) groups (Table-2).

### VLDL

There was no significant difference in VLDL concentration in the group treated with COQ10(30mg/kg) as compared with the control group. Groups treated with diclofenac (1mg/kg) and diclofenac (2mg/kg) showed a significant increase in serum VLDL concentration as compared with the control and COQ10(30mg/kg) groups. Furthermore, we observed a significant decrease in serum VLDL concentration in the group treated with diclofenac (1mg/kg) + COQ10(30mg/kg) in comparison with groups treated with diclofenac (1mg/kg) and diclofenac (2mg/kg) (Table-2).

### Serum urea

All the treated groups (COQ10(30mg/kg), diclofenac (1mg/kg), diclofenac (2mg/kg), diclofenac (1mg/kg) + COQ10(30mg/kg), and diclofenac (2mg/kg) + COQ10(30mg/kg) showed an increase in serum urea concentration when compared with the control group. The diclofenac groups (1mg/kg and 2mg/kg) had an increase in urea concentration in a dose-dependent manner, while the group treated with diclofenac (2mg/kg) + COQ10(30mg/kg) had a significant increase in urea concentration when compared with all other groups (Table-3).

**Table-3:** Effect of COQ10 and diclofenac on serum urea, creatinine, sodium, potassium, and chloride (n=8).

Group	Urea (mg/dl)	Creatinine (mg/dl)	Sodium (meq)	Potassium (meq)	Chloride (meq)
Control	3.58±0.18^a^	0.79±0.04^a^	135.81±2.19^a^	5.90±0.11^a^	102.37±1.77^a^
COQ10 (30 mg/kg)	4.27±0.13^b^	1.29±0.05^b^	146.62±2.61^b^	6.75±0.11^b^	114.12±1.20^b^
Diclofenac (1 mg/kg)	4.38±0.12^b^	1.32±0.05^b,c^	164.75±1.22^c^	8.55±0.13^c^	124.50±0.92^c^
Diclofenac (2 mg/kg)	4.87±0.14^c^	1.36±0.03^b,c^	177.12±1.70^d^	8.87±0.18^c^	137.62±1.61^d^
Diclofenac (1 mg/kg) +COQ10 (30 mg/kg)	5.01±0.11^c^	1.44±0.05^b,c^	177.62±1.77^d^	8.70±0.28^c^	135.75±1.30^d^
Diclofenac (2 mg/kg) +COQ10 (30 mg/kg)	6.63±0.13^d^	1.45±0.05^c^	181.25±3.56^d^	9.00±0.29^c^	138.87±1.44^d^

Values in each column followed by different superscript letters are significantly different at 5% level of significance

### Serum creatinine

There was a significant increase in serum creatinine concentration in samples from birds of the groups treated with COQ10(30mg/kg), diclofenac (1mg/kg), diclofenac (2mg/kg), diclofenac (1mg/kg) + COQ10(30mg/kg), and diclofenac (2mg/kg) + COQ10(30mg/kg) as compared to the control group. The group treated with diclofenac (2mg/kg) + COQ10(30mg/kg) had a significant increase in creatinine concentration when compared with the group that treated with COQ10(30mg/kg) (Table-3).

### Serum sodium

We observed a significant increase in serum sodium concentration in the groups that treated with COQ10(30mg/kg), diclofenac (1mg/kg), diclofenac (2mg/kg), diclofenac (1mg/kg) + COQ10(30mg/kg), and diclofenac (2mg/kg) + COQ10(30mg/kg) as compared to the control group. The diclofenac groups (1mg/kg and 2mg/kg) had an increase in sodium concentration in a dose-dependent manner. There was also a significant increase in serum sodium concentration in the groups treated with diclofenac (1mg/kg), diclofenac (2mg/kg), diclofenac (1mg/kg) + COQ10(30mg/kg), and diclofenac (2mg/kg) + COQ10(30mg/kg) as compared to the group treated with COQ10(30mg/kg) (Table-3).

### Serum potassium

All the treated groups (COQ10(30mg/kg), diclofenac (1mg/kg), diclofenac (2mg/kg), diclofenac (1mg/kg) + COQ10(30mg/kg), and diclofenac (2mg/kg) + COQ10(30mg/kg)) showed an increase in serum potassium concentration when compared with the control group. In addition, there was a significant increase in serum potassium concentration in the groups treated with diclofenac (1mg/kg), diclofenac (2mg/kg), diclofenac (1mg/kg) + COQ10(30mg/kg), and diclofenac (2mg/kg) + COQ10(30mg/kg) when compared to the COQ10(30mg/kg) treated group (Table-3).

### Serum chloride

There was a significant increase in serum chloride concentration among groupsCOQ10(30mg/kg), diclofenac (1mg/kg), diclofenac (2mg/kg), diclofenac (1mg/kg) + COQ10(30mg/kg), and diclofenac (2mg/kg) + COQ10(30mg/kg) when compared with the control group. Diclofenac groups (1mg/kg and 2mg/kg) had an increase in chloride concentration in a dose-dependent manner. Furthermore, there was a significant increase in serum chloride concentration in the groups which were treated with diclofenac (1mg/kg), diclofenac (2mg/kg), diclofenac (1mg/kg) + COQ10(30mg/kg), and diclofenac (2mg/kg) + COQ10(30mg/kg) as compared to the group treated with COQ10(30mg/kg) (Table-3).

## Discussion

In this study, diclofenac caused a significant increase in ALT and AST activity in a dose-dependent manner, an indicator of hepatorenal injury, which is in agreement with the findings of Saran who referred to an increase in AST and ALT activity in broiler chickens treated with diclofenac [13].

NSAIDs have been implicated as a cause of liver damage. Diclofenac is usually more related to hepatotoxicity [14]. Oxidative stress has an important responsibility in the pathophysiology of tissue damage. Similarly, diclofenac has been shown to induce oxidative injury in the liver [15]. Diclofenac causes bioactivation to reactive intermediate metabolites 4-OH and 5-OH diclofenac, following its metabolism by CYP2C9 and CYP3A4 enzymes [16]. Diclofenac metabolites can cause apoptosis of hepatocytes [17]. Lar revealed that liver injury in rats treated with diclofenac was characterized by hepatocellular degeneration, necrosis, blood vessel dilatation, lobules congestion, portal areas enlargement, and access of mixed inflammatory cells in the region of the necrotic hepatocytes and the portal area [18].

Mitochondria are fundamental organelles in cell homeostasis. Therefore, it is a possible target of drug-induced toxicity [19]. Mitochondrial malfunction is the key event in the pathogenic cascade, leading to ischemia-induced cell death from both necrosis and apoptosis [20]. Taha *et al*. [21] observed ultrastructure morphological alterations of mitochondrial shape represented by swelling, pleomorphic, destruction of their cristae, and condensation of its matrix induced by diclofenac treatment. Liver cell injury involves damage to the lysosomal membrane and liberation of proteolytic enzymes such as cathepsins (B, D, and L), which potentiates the opening of mitochondrial permeability transition (MPT) pore and cytochrome C liberation. This process then initiates events that activate caspase-3 and apoptosis [22].

The present study showed a significant decrease in ALT activity in groups treated with COQ10 and diclofenac together. This result indicates that COQ10 could repulse the liver damage from diclofenac; this action may be due to the antioxidant effect of COQ10. COQ10 has antioxidant activity that protects the phospholipids membrane and the mitochondrial membrane protein from oxidative damage induced by free radicals [23].

We noticed that COQ10 reduced the concentration of total cholesterol and LDL. However, it was not significant when compared to the control group. COQ10 has beneficial effects, especially on the harmful LDL. This effect can be attributed to the mechanism of action in reducing the production of cholesterol through a reduction of the enzymatic activity of 3-hydroxy-3-methylglutaryl coenzyme A reductases (HMGRs) in the liver [24]. The levels of HDL and triglyceride were not influenced by the treatment with COQ10 in agreement with the study by Gopi [25].

Diclofenac has a harmful effect on the liver, as discussed above, which leads to a significant alteration in total cholesterol, triglyceride, HDL, LDL, and VLDL. These findings are in agreement with those of Baravalia *et al*. [26], observed a noticeable increase in serum triglycerides and serum cholesterol in diclofenac-treated rats. Drug-induced hepatotoxicity has been shown to be linked with high levels of cholesterol and triglycerides [27]. This rise in the serum levels of total cholesterol and triglycerides can lead to hepatobiliary disorders and impaired cholesterol metabolism [28]. This effect is due to a dearth of lipoproteins, which halts triglyceride transport, causing them to stay in the hepatocytes and finally causing cellular lysis [29].

The adverse effects of diclofenac on the lipid profile have been alleviated by COQ10. While total cholesterol, triglyceride, HDL, and VLDL did not return to the normal levels, this may be due to the short treatment period (5 consecutive days). Importantly, there was a reduction in harmful LDL level below the level of the control group. COQ10 has shown to induce a reduction in serum LDL cholesterol levels in hyperlipidemic rabbits [30]. This effect of COQ10 is consistent with antioxidant behavior, which protects LDL from free radicals and oxidative damage [23]. This result gives a good impression of COQ10 effectiveness in reduction of the detrimental effects of diclofenac on the lipid profile.

COQ10 caused a negative effect on the kidneys when administered alone; but when coadministered with diclofenac, COQ10 leads to a synergistic detrimental effect on the kidneys. Diclofenac induces nephrotoxicity, represented by a rise in the levels of urea, creatinine, and electrolytes (Na, K, and Cl). Toxic doses of diclofenac can cause nephrotoxicity in experimental animals and humans [31].

We observed an increase in the levels of urea and creatinine in groups treated with diclofenac. This result is in agreement with several studies; observed increases in urea and creatinine concentration in the following animals treated with diclofenac: Broiler chickens [13], rats [31], rabbits [32], and mice [33]. Levels of serum electrolytes (Na+, K+, and Cl−) are good markers of kidney function. Elevations in levels of serum electrolytes Na, K, and Cl observed in this study could be the cause of the harmful effect of diclofenac on the kidneys. This result is in agreement with a study by Orinya [34].

NSAIDs block prostaglandin synthesis by inhibiting the COX enzyme. There are two isoforms of COX (COX-1 and COX-2); COX-1 controls the glomerular filtration rate (GFR) and renal hemodynamics while COX-2 controls salt and water excretion[2]. Prostaglandins are powerful vasodilators; they are essential in the maintenance of blood flow to the kidneys, as well as having a central role in the intrarenal blood flow distribution [35]. The inhibition of prostaglandin synthesis from arachidonic acid leads to vasoconstriction and a decrease in blood flow with a decline in glomerular capillary pressure, consequential in a rapid decrease in glomerular filtration rate[36]. Imamura used contrast-enhanced ultrasonography to declare that diclofenac reduces renal blood flow [37]. NSAIDs inhibit prostaglandin formation, leading to the appearance of kidney-related adverse effects such as increased urea and creatinine levels [38]. Various forms of kidney failure have been reported in connection with NSAIDs use, including severe fall of nephritic function, severe interstitial nephritis, nephritic papillary necrosis, hyperkalemia, and sodium plus fluid retention [39].

In this study, there was a significant increase in AST activity; this result is compatible with Fathi [40], who referred to a slight increase in AST after treatment with COQ10 in broiler chickens at doses of 20 and 40mg/kg. COQ10 causes an elevation in the levels of urea, creatinine, and electrolytes (Na, K, and Cl). This effect indicates impairment in the kidney function, which is somewhat unusual. This directed us to review the references to ascertain an explanation illustrating this effect. Civenni illustrated inhibition of arachidonic acid metabolism through COQ10 in astrocyte homogenates [41]. We developed a hypothesis on this outcome that COQ10 can inhibit prostaglandin synthesis in the kidney; this effect is similar to the action of NSAIDs. In addition, COQ10 can inhibit nitric oxide (NO) formation [42]. NO is a classic vasodilator agent. It maintains an effective balance with the vasoconstrictive agents in physiological conditions; thus, the blood vessels over contract when NO secretion suppresses [43]. This explanation indicates another theory about COQ10’s effect on the kidneys, which leads to increased impairment in kidney function. In this study, when diclofenac was coadministered with COQ10, urea increased and electrolytes increased slightly due to the synergistic effects of both compounds.

## Conclusion

Diclofenac causes liver function disturbance manifested by an increase in ALT and AST and an increase in the lipid profile parameters. COQ10 can antagonize this harmful effect of diclofenac on the liver of broiler chickens. Our study also shows that diclofenac and COQ10 administered together lead to kidney function disturbance, represented by an increase in urea, creatinine, and electrolytes (Na, K, and Cl). Our study confirmed that administering diclofenac and COQ10 together lead to synergistic harmful effects of both compounds on the kidneys and increase renal impairment. Further studies are necessary to confirm our findings.

## Authors’ Contributions

Both authors worked equally and approved the final manuscript.
